# Demographic and Clinical Characteristics of Discharge Against Medical Advice (DAMA) in Emergency Departments of Madinah Hospitals: A Retrospective Cross-Sectional Study

**DOI:** 10.7759/cureus.105704

**Published:** 2026-03-23

**Authors:** Khalid A Ateyyah, Ahmad Z Halawani, Nosaiba S Alharbi, Najwa A Alqurashi, Ranad S Alamri, Abdullah A Najjar, Amirah F Alzughaibi, Mohammad W Aljayyar, Aseel O Alsubhi

**Affiliations:** 1 Emergency Medicine, Taibah University, Madinah, SAU; 2 Emergency Medicine, Madinah Health Cluster, King Fahad Hospital, Madinah, SAU; 3 Emergency Medicine, King Abdulaziz Medical City, Riyadh, SAU; 4 Emergency Medicine, King Saud Medical City, Riyadh, SAU; 5 Emergency Medicine, Ministry of Health Holdings, Madinah, SAU; 6 General Surgery, King Salman Specialist Hospital, Hail, SAU; 7 Emergency Medicine, Saudi German Hospital, Jeddah, SAU; 8 General Surgery, King Salman Bin Abdulaziz Medical City, Madinah, SAU; 9 Medicine, Taibah University, Madinah, SAU

**Keywords:** dama, emergency departments, healthcare utilization, patient demographics, saudi arabia

## Abstract

Background

Discharge against medical advice (DAMA) is a significant global healthcare challenge, occurring when a patient or caregiver, in pediatric cases, leaves the hospital against medical recommendations. DAMA can lead to adverse health outcomes, increased hospital readmissions, and strain on healthcare resources. This study examines the characteristics of DAMA in emergency departments (EDs) at Madinah hospitals in Saudi Arabia and explores its distribution according to demographic factors.

Methodology

A retrospective cross-sectional study was conducted using secondary quantitative data from hospital records at King Fahad Hospital and King Salman Bin Abdulaziz Medical City in Madinah, Saudi Arabia. Ethical approval was obtained, and descriptive statistical analyses were performed to assess demographic trends and reasons for DAMA.

Results

Starting from January to December 2022, a total of 1,120 DAMA cases were recorded. The highest proportion of cases was observed among middle-aged adults (30-49 years), with males accounting for 57.5% (n = 645) of cases. DAMA was more frequent on weekdays, peaking on Sundays, and most cases occurred in the afternoon and night shifts. A large proportion of patients (59.1%, n = 663) did not provide a reason for DAMA, while 28.7% (n = 322) refused admission, 4.6% (n = 52) cited long wait times, 1.7% (n = 20) had financial constraints, and 2.5% (n = 29) left after feeling better.

Conclusion

DAMA remains a critical concern in Saudi Arabian EDs, with a higher proportion of recorded cases among males and middle-aged adults. Contributing factors include refusal of medical care, long wait times, financial limitations, and perceived early recovery. The high prevalence of DAMA during peak hours suggests systemic challenges in hospital resource management. Addressing these issues requires improved patient education, streamlined hospital processes, and enhanced communication strategies to reduce DAMA rates and improve patient outcomes.

## Introduction

Healthcare delivery excellence is determined not only by medical treatment results but also by patient devotion toward recommended care. When patients leave the hospital before completion of treatment, it compromises system efficiency and the patient’s health. Premature discharge from the hospital results in complications and an increase in readmissions, with an unnecessary increment of healthcare costs and bills that take place on hospital resources and personal expense. In this way, emergency departments (EDs) play a critical role in saving lives, often serving as an indicator of the overall quality of care provided by a hospital, particularly for critically ill patients [1]. Discharge against medical advice (DAMA) is recognized as a significant global concern in healthcare, as noted by Ramakrishnan et al. [1]. DAMA occurs when a patient leaves a hospital or medical facility before receiving formal discharge approval from their attending physician, potentially leading to adverse consequences for both the patient and the healthcare system. The frequency of DAMA varies widely, with studies reporting DAMA rates ranging from 1% to 2%, while some studies have documented rates as high as 25% [2-5]. Specifically, among hospitalized patients, DAMA rates range from 0.7% to 2.2% in EDs and approximately 0.9% overall [6,7].

Physicians across specialties frequently encounter patients who refuse medical assistance and insist on leaving against medical advice. This situation presents ethical and professional challenges, as physicians must balance respect for patient autonomy with concerns about incomplete treatment and potential health risks [8-10]. Sudden discontinuation of medical treatment, whether by the patient or their guardians, can lead to complications, readmission, or even mortality. DAMA has been associated with increased healthcare burdens. The report by El-Metwally et al. found that patients discharged against medical advice have a 28% higher risk of readmission within a month for the same or a related condition, along with an 8% higher risk of subsequent hospital visits, resulting in additional medical costs [4]. Many studies identified that the most common reasons for DAMA include perceived symptom relief, partial recovery, and distrust in hospital care quality [11,12]. Additional studies have found that family obligations, financial constraints, long ED wait times, and overcrowding are key factors influencing DAMA decisions [13].

Addressing DAMA requires targeted interventions, including enhanced patient counseling by nurses and paramedics about the potential risks of premature discharge. Training programs should also focus on educating patient companions, especially in cases involving children and women, where informed consent is often required for medical decisions [14,15]. Furthermore, Alwallan et al. noted that DAMA rates in developing countries are approximately twice as high as in developed nations, largely due to financial constraints and the absence of comprehensive healthcare systems [16]. Understanding the determinants of DAMA is essential for developing effective strategies to mitigate its impact. This study aims to assess the characteristics of DAMA in hospital EDs in Madinah and to explore documented factors influencing patients' decisions to leave against medical advice. Focusing on these factors can help in the development of targeted interventions to improve emergency care outcomes and patient care.

## Materials and methods

Study design and settings

This study employed a retrospective cross-sectional research design to examine the distribution of DAMA. It uses secondary quantitative data collection methods, as described by El-Metwally et al. [4]. An explanatory research approach was adopted to address gaps in understanding the characteristics of DAMA. This was a retrospective, descriptive cross-sectional study based on hospital record review of all recorded DAMA cases identified during the study period [17].

Data collection

The data for this study were obtained from the hospital records of King Fahad Hospital and King Salman Bin Abdulaziz Medical City in Madinah, Saudi Arabia. As this was a retrospective chart review based on hospital records, the requirement for informed consent was waived by the Institutional Review Board. The study focused on patients admitted to the EDs of these hospitals between January 1, 2022, and December 31, 2022. All recorded DAMA cases during the study period were eligible for inclusion regardless of age; therefore, pediatric patients were included when applicable. The extracted patient information included: demographic details (gender, age at admission, and nationality), clinical and admission details (mode of arrival, triage category, and hospital stay duration), and discharge details (date and time of discharge). As the study included only DAMA cases and lacked a comparison group of non-DAMA patients, these variables were used to describe the characteristics of DAMA cases rather than to identify risk factors or determinants of DAMA. Data were manually extracted by seven research team members, who entered patient records using a standardized data collection form created in Google Forms (Google, Inc., Mountain View, CA), which were subsequently transferred to Excel (Microsoft Corp., Redmond, WA) for further processing and analysis. Each recorded DAMA encounter was treated as a separate case in the analysis; therefore, repeat DAMA visits by the same patient during the study period were counted as separate records.

Ethical considerations

Ethical approval was attained from the Institutional Review Board of the College of Medicine, Taibah University, Madinah, Saudi Arabia (IRB approval no.: H-03-M-84; log no.: 24-045). The requirement for individual informed consent was waived by the Institutional Review Board because this study was a retrospective review of anonymized hospital records. Access to hospital records was granted following formal approval from hospital management, supported by an official letter from the university and ethical clearance documentation. 

Data analysis

A quantitative data collection approach was attained, similar to that of Noohi et al. [18]. A non-probability sampling method was used to select patient records containing relevant information, focusing on understanding the occurrence and determinants of DAMA in Madinah hospitals. The collected data were analyzed using descriptive and demographic statistical methods to determine the distribution of DAMA in Madinah hospitals [19]. Secondary data analysis was conducted to identify key trends and patterns related to DAMA cases in Saudi Arabia, with a specific focus on Madinah hospitals. Descriptive statistics were used to summarize the characteristics of the study population. Categorical variables, such as gender, nationality, triage level, chief complaints, DAMA reasons, and frequency, were presented as frequencies (N) and percentages (%). Continuous variables, such as age and length of stay, were expressed as means and standard deviations (SDs). All data were entered and analyzed using IBM SPSS Statistics version 27.0.1 (IBM Corp., Armonk, NY). Data cleaning and verification were performed before analysis to ensure accuracy and completeness. Proportions were presented with 95% confidence intervals. Descriptive subgroup analyses were also performed according to age group, sex, nationality, day of visit, and time of ED visit.

## Results

Demographic and clinical characteristics of ED patients with DAMA

The dataset comprises information on 1,120 ED patients who were DAMA. The age distribution reveals that the largest proportion of patients was in the age group of 30-39 years (N = 206, 18.4%), followed closely by those aged 20-29 years (N = 194, 17.3%) and 40-49 years (N = 146, 13.0%). Very young children aged 0-9 years accounted for 4.2% (N = 47), while the elderly population aged 70 years and above collectively represented a smaller segment, with those aged 70-79 years comprising 8.6% (N = 96), and only a few patients aged 90 years and above (N = 7, 0.6%). In terms of gender, males accounted for 57.6% of DAMA cases (645/1120; 95% CI: 54.7%-60.5%), while females accounted for 42.4% (475/1120; 95% CI: 39.5%-45.3%). Saudi nationals represented 69.2% of DAMA cases (775/1120; 95% CI: 66.5%-71.9%), whereas non-Saudi patients accounted for 30.8% (345/1120; 95% CI: 28.1%-33.5%). Triage levels at presentation indicated that most patients were categorized as level 3 (N = 771, 68.8%), followed by level 4 (N = 183, 16.3%), while critical cases (triage level 1) were relatively rare (N = 67, 6.0%). The ED visits were more frequent on weekdays (N = 783, 69.9%) than on weekends (N = 337, 30.1%). The distribution across individual days showed a fairly even spread, with Sunday recording the highest attendance (N = 184, 16.4%) and Thursday the lowest (N = 141, 12.6%). Regarding the time of ED visits, the afternoon shift was the most common (N = 397, 35.4%), followed by the night shift (N = 368, 32.9%) and the morning shift (N = 355, 31.7%). The average length of stay in the ED was 9.07 hours, with a standard deviation of 11.07 hours (Table 1).

**Table 1:** Demographic and clinical characteristics of emergency department visitors

		N	%
Age	0-9	47	4.2%
	10-19	90	8.0%
	20-29	194	17.3%
	30-39	206	18.4%
	40-49	146	13.0%
	50-59	144	12.9%
	60-69	146	13.0%
	70-79	96	8.6%
	80-89	44	3.9%
	90-99	4	0.4%
	100-110	3	0.3%
Gender	Female	475	42.4%
	Male	645	57.6%
Nationality	Non-Saudi	345	30.8%
	Saudi	775	69.2%
Triage level	1	67	6.0%
	2	74	6.6%
	3	771	68.8%
	4	183	16.3%
	5	25	2.2%
Day of visit	Weekday	783	69.9%
	Weekend	337	30.1%
Day	Monday	161	14.4%
	Tuesday	168	15.0%
	Wednesday	143	12.8%
	Thursday	141	12.6%
	Friday	165	14.7%
	Saturday	158	14.1%
	Sunday	184	16.4%
Time of ED visit	Morning shift	355	31.7%
	Afternoon shift	397	35.4%
	Night shift	368	32.9%
Length of stay		9.07±11.07 hours	

Distribution of chief complaints among ED visitors

The analysis of chief complaints among ED visitors reveals that abdominal pain was the most frequently reported issue, accounting for 167 cases (14.9%). A significant portion of visits were recorded without a clearly documented reason, marked as "unknown complaints" (N = 142, 12.7%). Chest pain was also a common presenting complaint (N = 112, 10.0%), followed closely by extremity trauma (N = 104, 9.3%) and shortness of breath (N = 85, 7.6%). Less frequent but still notable were cases involving multiple trauma (N = 49, 4.4%), musculoskeletal pain (N = 37, 3.3%), dizziness or vertigo (N = 36, 3.2%), weakness (N = 35, 3.1%), and seizures (N = 27, 2.4%). Other symptoms such as fever and/or rigors (N = 26, 2.3%), nausea and/or vomiting (N = 25, 2.2%), headache (N = 22, 2.0%), and head trauma (N = 22, 2.0%) were also documented. A range of less common complaints each contributed to less than 2% of visits, including cough (N = 17, 1.5%), skin rash or lesion (N = 15, 1.3%), loss of consciousness (N = 14, 1.3%), and flank pain (N = 12, 1.1%). Road traffic accidents represented 1.0% of cases (N = 11), while other causes such as venomous animal injuries or bites, altered mental status, and unilateral leg swelling each accounted for 0.9% or less. A wide variety of other symptoms, including thoracic trauma, testicular pain or swelling, visual disturbances, hypoglycemia, hemoptysis, fatigue, falls, diabetic foot, and various forms of abdominal trauma, were reported in small numbers, each typically comprising between 0.3% and 0.4% of total cases (N = 3 to 5). The remaining complaints, such as vaginal bleeding, slurred speech, dyspnea, anal pain, abnormal mood, and others, each occurred in only one or two patients, representing 0.1% to 0.2% of visits. Overall, the data reflect a broad spectrum of clinical presentations, with abdominal and chest complaints, trauma-related injuries, and non-specific symptoms such as dizziness and weakness comprising a substantial portion of ED utilization. Rare and less specific symptoms contributed minimally to the total ED caseload (Table 2). Subgroup analysis based on day of visit demonstrated that DAMA cases were more frequently recorded on weekdays (69.9%) than on weekends (30.1%). Among individual days, Sunday accounted for the highest proportion of cases (16.4%), followed by Tuesday (15.0%), Friday (14.7%), Monday (14.3%), Saturday (14.1%), Wednesday (12.7%), and Thursday (12.5%). Subgroup analysis based on time of ED visit showed that the afternoon shift (12 PM-6 PM) accounted for the largest proportion of DAMA cases (35.4%), followed by the night shift (6 PM-12 AM, 32.8%) and the morning shift (6 AM-12 PM, 31.6%).

**Table 2:** Distribution of chief complaints among emergency department visitors

		N	%
Chief complain	Abdominal pain	167	14.9%
	Unknown complaints	142	12.7%
	Chest pain	112	10.0%
	Extremity trauma	104	9.3%
	Shortness of breath	85	7.6%
	Multiple trauma	49	4.4%
	Musculoskeletal pain	37	3.3%
	Dizziness or vertigo	36	3.2%
	Weakness	35	3.1%
	Seizure	27	2.4%
	Fever and/or rigors	26	2.3%
	Nausea and/or vomiting	25	2.2%
	Headache	22	2.0%
	Head trauma	22	2.0%
	Cough	17	1.5%
	Skin rash or lesion	15	1.3%
	Loss of consciousness	14	1.3%
	Flank pain	12	1.1%
	Road traffic accident (RTA)	11	1.0%
	Venomous animal injury or mammalian bites	10	0.9%
	Altered mental status (acute confusion)	10	0.9%
	Unilateral leg swelling	9	0.8%
	Numbness or disturbed sensory function	8	0.7%
	Sore throat	7	0.6%
	Thoracic trauma	6	0.5%
	Testicular pain and/or swelling	6	0.5%
	Visual disturbance (including visual impairment and double vision)	5	0.4%
	Hypoglycemia	5	0.4%
	Hemoptysis	5	0.4%
	Fatigue	5	0.4%
	Fall down	5	0.4%
	Diabetic foot	5	0.4%
	Abdominal trauma	5	0.4%
	Thermal burns	4	0.4%
	Hematuria	4	0.4%
	Hematemesis	4	0.4%
	Foreign bodies (airway, ear, nasal, ingested, rectal, and vaginal)	4	0.4%
	Abdominal distention	4	0.4%
	Palpitation	3	0.3%
	Painful crisis (SCA)	3	0.3%
	Known intoxication	3	0.3%
	Hyperglycemia	3	0.3%
	Generalized body ache	3	0.3%
	Diarrhea	3	0.3%
	Abscess	3	0.3%
	Vaginal bleeding	2	0.2%
	Slurred speech	2	0.2%
	Melena	2	0.2%
	Ear pain	2	0.2%
	Dysuria	2	0.2%
	Dyspnea	2	0.2%
	Chemical exposure	2	0.2%
	Bilateral leg swelling	2	0.2%
	Anal pain	2	0.2%
	Abnormal mood	2	0.2%
	Abdominal swelling	2	0.2%
	Sepsis (SSI)	1	0.1%
	Pelvic pain	1	0.1%
	Hypotension	1	0.1%
	Facial trauma	1	0.1%
	Epistaxis	1	0.1%
	Dysphagia	1	0.1%
	Constipation	1	0.1%
	Chest trauma	1	0.1%

Characteristics, causes, and frequency of DAMA

The data on DAMA provide insights into the individuals involved in signing DAMA forms, the reported reasons behind DAMA decisions, and the frequency of such occurrences among patients. In more than half of the cases, DAMA was signed by the patients themselves (N = 586, 52.3%), while in a significant number of cases, the signer was not clearly documented (N = 399, 35.6%), indicating substantial missing documentation in hospital records. Guardians accounted for 122 signings (10.9%), with very few cases signed by a husband (N = 5, 0.4%), a relative (N = 3, 0.3%), a brother (N = 2, 0.2%), or a daughter (N = 1, 0.1%). In two cases (0.2%), DAMA was not signed at all. No documented reason was recorded for 59.2% of cases (663/1120; 95% CI: 56.3%-62.1%). Refusal of admission or medical care accounted for 28.7% (322/1120; 95% CI: 26.1%-31.4%). Long waiting time was reported in 4.6% (52/1120; 95% CI: 3.4%-5.9%), while 2.6% of patients left after feeling better (29/1120; 95% CI: 1.7%-3.5%). Financial reasons were documented in 1.8% (20/1120; 95% CI: 1.0%-2.6%). Subgroup analysis according to sex showed that refusal of admission or medical care was the most frequently documented reason for DAMA among both male and female patients. Less frequent reasons included dissatisfaction with care (N = 7, 0.6%), family concerns such as caring for children or other sick family members (N = 5, 0.4%), and delays in the availability of suitable services such as the operating room or catheterization lab (N = 4, 0.4%). A small number of patients absconded without formal DAMA (N = 3, 0.3%). Regarding the frequency of DAMA occurrences, the majority of patients had never left against medical advice (N = 629, 56.2%). A notable proportion reported leaving a few times (less than 3) (N = 338, 30.2%), while the frequency was unknown for 118 individuals (10.5%). Only 35 patients (3.1%) had a history of leaving against medical advice three or more times. These findings highlight the complexity and variety of factors influencing DAMA, with a substantial portion of cases lacking documentation of both the signer and the reason for discharge (Table 3).

**Table 3:** Characteristics, causes, and frequency of discharge against medical advice (DAMA)

		N	%
Who signed DAMA	Patient	586	52.3%
	Unknown	399	35.6%
	Guardians	122	10.9%
	Husband	5	0.4%
	Relative	3	0.3%
	Not signed	2	0.2%
	Brother	2	0.2%
	Daughter	1	0.1%
Cause of DAMA	No reason mentioned	663	59.2%
	Refusing admission/medical care	322	28.7%
	Long wait time	52	4.6%
	Feeling better	29	2.6%
	Financial reasons	20	1.8%
	Incomplete workup	15	1.3%
	Dissatisfaction with care	7	0.6%
	Family concerns (needs to take care of kids, have a sick family member)	5	0.4%
	Delay in availability of suitable services (OR, cath lab)	4	0.4%
	Absconded	3	0.3%
Frequency of DAMA	Never	629	56.2%
	A few time (less than 3)	338	30.2%
	Unknown	118	10.5%
	Many times (3 and more)	35	3.1%

## Discussion

DAMA is a significant challenge in healthcare management worldwide, contributing to increased healthcare expenditures, system inefficiencies, and adverse patient outcomes, including mortality. Despite extensive research on ED overcrowding, there remains a paucity of studies investigating the patterns of DAMA and their impact on patient health and healthcare systems [8]. DAMA presents an ethical dilemma for healthcare providers, as it involves discharging patients before completing necessary treatment, raising concerns among physicians regarding patient safety and treatment efficacy [10,20,21]. Patients who opt for DAMA must provide signed consent, yet this practice is associated with increased readmission rates and, in severe cases, heightened mortality risks due to incomplete medical care. Given that ED patients often present with critical conditions, understanding the etiological and prognostic factors of DAMA is essential to mitigate its occurrence and improve patient outcomes [10,21].

The findings indicate that a higher proportion of DAMA cases occurred among middle-aged adults (30-49 years), followed by those aged 50-69 years and 10-29 years. This trend may reflect work-related commitments, personal responsibilities, and perceptions of manageable health conditions, which may drive DAMA decisions among this demographic [22]. Conversely, lower DAMA rates among children (zero to nine years) and elderly patients (70-89 years) may reflect increased parental or caregiver involvement in healthcare decisions and greater medical attention provided to older adults due to their vulnerability [23].

There is a higher proportion of DAMA cases among Saudi nationals, with males accounting for a higher rate of DAMA cases (57.5%) than females (42.4%). This aligns with previous studies conducted in Saudi Arabia, which have suggested that professional and societal obligations may contribute to men prioritizing external commitments over hospitalization [24]. The higher proportion of DAMA cases among Saudi nationals (69.1%) may reflect cultural norms, familiarity with the healthcare system, and possibly greater confidence in seeking alternative medical care [13]. The temporal distribution of DAMA cases revealed a higher frequency on weekdays, with Sunday accounting for the highest proportion of cases. This pattern may be related to increased hospital visits at the start of the workweek, which could contribute to higher patient volume and hospital resource strain [25]. Additionally, DAMA was more frequent during the afternoon shift (12 PM-6 PM), followed by the night shift (6 PM-12 AM), suggesting a possible association with long waiting times, fatigue, and personal obligations in the later hours of the day [26]. Subgroup analyses further highlighted variations in the distribution of DAMA cases across age group, sex, nationality, day of visit, and time of ED visit, while confidence intervals improved the precision of the reported descriptive estimates.

A striking observation in this study is the high proportion of patients (63%) with "unknown complaints." Similarly, a substantial proportion of records lacked documentation of the DAMA signer and reasons for leaving. Such missing information may introduce information bias and limit the ability to fully interpret the factors contributing to DAMA decisions. Incomplete documentation in retrospective hospital records may lead to underestimation or misclassification of the true reasons for DAMA.

This raises concerns about the quality of medical documentation and may also reflect incomplete recording of patient evaluations before DAMA. Inadequate documentation may reflect poor communication between patients and healthcare providers, which could contribute to uncertainty about medical care and underestimation of illness severity [27]. Among documented diagnoses, abdominal pain (14.9%) and chest pain (10%) were the most common presenting complaints. These findings align with previous studies that identified similar trends, suggesting that some patients with apparently less severe conditions may choose to leave the hospital prematurely. Patients experiencing musculoskeletal pain, vertigo, weakness, seizures, fever, headache, and other minor ailments may perceive their conditions as manageable without hospital admission [28,29].

Study significance

Understanding the characteristics and documented reasons contributing to DAMA is crucial for improving patient care and optimizing healthcare system efficiency. This study provides valuable insights into the prevalence, demographic and clinical characteristics, and underlying causes of DAMA in EDs, particularly within the Saudi Arabian healthcare context. By identifying key trends, such as age and gender disparities, common presenting complaints, and documentation gaps, this research highlights areas for targeted interventions. The findings emphasize the need for improved patient education, enhanced medical record-keeping, and system-level strategies to reduce DAMA rates. Moreover, the study contributes to the global literature by offering region-specific data that can inform policy decisions, hospital management strategies, and future research aimed at mitigating the risks associated with premature discharge.

Limitations

This study has several limitations that should be acknowledged. A major limitation of this study is the high proportion of missing data in several variables, including undocumented reasons for DAMA and unidentified signers of the DAMA form. A significant proportion of DAMA cases had incomplete documentation, with 63% recorded as having "unknown complaints" and 59.1% lacking stated reasons for discharge, limiting the accuracy of clinical assessments and underlying motivations for DAMA. This incomplete documentation introduces potential information bias and may have influenced the accuracy of reported reasons for DAMA. As a result, the reported distributions of DAMA reasons should be interpreted with caution.

The study did not include the total number of ED visits during the study period; therefore, a true prevalence of DAMA could not be calculated. In addition, only descriptive statistics were performed, and no inferential analysis was conducted to identify independent predictors or determinants of DAMA. The absence of a comparison group of non-DAMA patients further limited the ability to identify risk factors, predictors, or determinants of DAMA. Time-specific trends were identified, but the study did not establish causal links between hospital workflow, patient satisfaction, and DAMA occurrences. In addition, the descriptive nature of the analysis limits causal interpretation, and the explanations discussed should be considered hypothesis-generating rather than confirmatory.

Additionally, the study was conducted at two hospitals in Madinah, Saudi Arabia, which may restrict the generalizability of findings to other regions. The absence of follow-up data prevents assessment of long-term health outcomes and readmission rates among DAMA patients. Financial barriers to care may also be underreported, as only 1.7% of patients cited economic reasons for leaving against medical advice. Furthermore, while cultural and social factors likely influence DAMA decisions, this study did not include qualitative analyses to explore these aspects in depth. Addressing these limitations in future research will help improve data quality, enhance understanding of DAMA characteristics, and guide the development of targeted interventions.

## Conclusions

This study highlights the characteristics, demographic patterns, temporal trends, and underlying reasons for DAMA at two major hospitals in Madinah, Saudi Arabia. The findings indicate that a higher proportion of recorded DAMA cases occurred among middle-aged adults, particularly males and Saudi nationals, suggesting that work-related commitments, cultural factors, and perceived illness severity may influence premature discharge decisions. Temporal patterns reveal a higher incidence on weekdays, particularly during afternoon and night shifts, emphasizing the role of hospital workflow and patient obligations in DAMA occurrences. The high proportion of cases with unknown complaints and undocumented reasons for DAMA raises concerns about the accuracy of medical records and patient-provider communication. Common diagnoses, including abdominal and chest pain, suggest that many DAMA cases involve non-life-threatening conditions, potentially leading patients to underestimate the risks of leaving against medical advice. Addressing documentation gaps, improving hospital efficiency, and enhancing patient education on DAMA-associated risks are crucial steps toward reducing DAMA rates and improving patient outcomes. Future research should focus on qualitative analyses and follow-up assessments to gain deeper insights into patient motivations and the long-term health consequences of DAMA.
